# A rare presentation of hepatolithiasis in an adolescent patient: A case report

**DOI:** 10.1016/j.ijscr.2020.06.017

**Published:** 2020-06-12

**Authors:** Jonathan Freise, Jorge Mena, Kwun Wah Wen, Marshall Stoller, Sunita Ho, Carlos Corvera

**Affiliations:** a School of Medicine, University of California San Francisco, San Francisco, CA, USA; b Department of Pathology, University of California San Francisco, San Francisco, CA, USA; c Department of Urology, University of California San Francisco, San Francisco, CA, USA; d Department of Preventive and Restorative Dental Sciences, Division of Biomaterials and Bioengineering, University of California San Francisco, San Francisco, CA, USA; e Department of Surgery, Division of Surgical Oncology, University of California San Francisco, San Francisco, CA, USA

**Keywords:** EDX, Electron Dispersive X-Ray, Micro-XCT, Micro X-Ray Computerized Tomography, MRCP, magnetic resonance cholangiopancreatography, PSC, primary sclerosing cholangitis, SEM, Scanning Electron Microscopy, Adolescent, Case report, Hepatic duct, Hepatolithiasis, Intrahepatic stone

## Abstract

**Introduction:**

Hepatolithiasis (intrahepatic stones) is rare in adolescent patients and requires complex management strategies to prevent recurrent infections and progression to hepatic fibrosis. Surgical management is often required. In cases of unclear etiology, further work-up is indicated to provide insight into future management. In this report we describe an extensive stone analysis.

**Presentation of case:**

A 20-year-old Caucasian female presented with known hepatolithiasis and multiple prior recurrent bouts of abdominal pain requiring hospitalization. Magnetic resonance cholangiopancreatography (MRCP) demonstrated an abnormal left-sided hepatic biliary ductal system dilatation. She was treated surgically with a formal left hepatectomy and preservation of the caudate lobe. The right ductal system had no stones or evidence of inflammation, and her bile and stones cultures were negative for organism growth. An extensive analysis demonstrated stone composition primarily of cholesterol.

**Discussion:**

Adolescent presentations of hepatolithiasis are rare and considerations in the differential diagnosis include primary sclerosing cholangitis, bile acid transporter defects, and other known genetic diseases. This case is unique because only the left half of the intrahepatic ductal system had evidence of stone disease and the bile was sterile. A detailed stone analysis demonstrating cholesterol supersaturation provides additional context though the etiology remains unclear in this case and will require lifelong follow-up.

**Conclusion:**

Early-onset hepatolithiasis is rare and requires expert management, and in some cases definitive surgical management with life-long follow-up. Extensive stone analysis and genetic testing can be performed to help identify disease etiology in unique cases.

## 1 Introduction

Hepatolithiasis is defined as the presence of gallstones in the bile ducts proximal to the confluence of the right and left hepatic ducts, irrespective of the co-existence of stone disease in the common bile duct and gallbladder. The prevalence of hepatolithiasis is highest in East Asia and rare in Western countries [1]. Moreover, incidence occurs most commonly in the fifth and sixth decades, though symptoms of the intrahepatic type are more frequent in younger age groups [2]. Etiology remains unknown in most cases, although genetic and environmental factors are thought to contribute. Stones are classified by composition into calcium bilirubinate stones and cholesterol stones, with 75% of cases being calcium bilirubinate [3]. Treatment goals focus on resolving recurrent infection and preventing subsequent hepatic fibrosis and progression to cholangiocarcinoma. The most efficacious treatment to date has been surgery, including removal of affected liver segment(s) to prevent recurrence of stone formation and progressive hepatocellular injury. In this case report, we discuss a unique presentation of hepatolithiasis at our academic center with a comprehensive work-up of the patient’s stone disease to better characterize etiology. This work is reported according to the SCARE and PROCESS criteria [4,5].

## 2 Presentation of case

A 20-year-old Caucasian female with a strong family history of early onset gallbladder disease first presented to our center with known hepatolithiasis and recurrent bouts of severe abdominal pain requiring multiple hospitalizations. She was first hospitalized at the age of 13 for right upper quadrant abdominal pain found to have choledocholithiasis with intrahepatic and extrahepatic biliary duct dilatation. She underwent endoscopic retrograde cholangiopancreatography during her admission had a subsequent cholecystectomy with no evidence of cholelithiasis. In the following years, she experienced recurrent bouts of severe abdominal pain without any fever or chills and multiple MRCP studies that demonstrated abnormal left hepatic biliary ductal system dilatation. At the time of her presentation to our center, she was noted to have a dominant stone impacted at the origin of her left main hepatic duct with a left sided dilated ductal system. She was taken to the operating room for a formal left hepatectomy. Intraoperatively, she was found to have palpable stone disease throughout her left liver with a large stone aggregate at the origin of the left hepatic duct. Choledochoscopy of the right sided ductal system demonstrated normal biliary epithelium without inflammation or stones. She was treated by formal left hepatectomy and preservation of her extrahepatic biliary tract. Her preoperative symptom of abdominal pain completely resolved, however, her recovery was complicated by the development of pruritus two weeks after discharge.

**Fig. 1:**
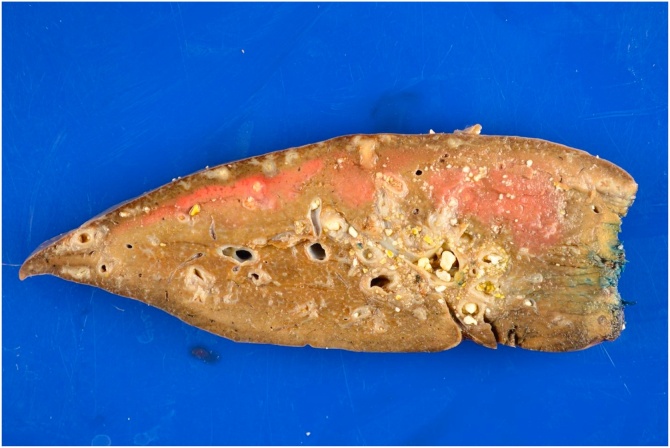
Hemisection of left liver lobe demonstrating yellow tan intrahepatic stones filling the dilated left biliary ductal system and fibrosis and inflammation of the bile duct walls.

An MRCP showed isolated and dilated biliary ducts from the caudate lobe with eventual resolution of her symptoms. Bile culture samples from the operative procedure were negative for organism growth. Pathology demonstrated numerous (up to 0.5 mm) yellow tan calculi filling the entire dilated left biliary ductal system and fibrosis and inflammation of the bile duct walls, as seen in the section of left liver lobe (Fig. 1). The hepatic parenchyma showed marked sinusoidal dilatation, congestion, and hepatic plate atrophy. No tumor or parasite was found. Calculi composition was noted to be primarily of cholesterol with some calcium bilirubinate, bile salts and pigments consistent with a bile stone. Given the unique presentation of the patient’s disease, a more extensive analysis was performed on the stones.

**Fig. 2:**
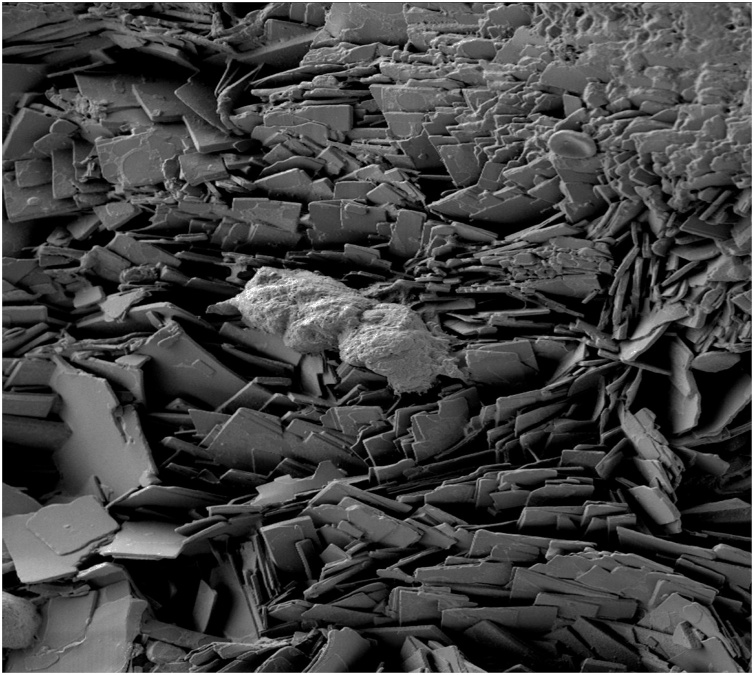
SEM of the surface of whole specimens and of the core of fractured specimens demonstrating stones composed almost entirely of smooth plates.

Material analysis of the stones was performed using Micro X-Ray Computerized Tomography (Micro-XCT) for density and internal structure, and Scanning Electron Microscopy (SEM) for ultrastructure. Elemental analysis was performed using Electron Dispersive X-Ray (EDX) Spectroscopy. Micro-XCT of stone cross section (not shown) demonstrated low mineral density throughout the specimen and a hollow core. SEM of the surface of whole specimens and of the core of fractured specimens demonstrated the stones were made almost entirely of smooth plates (Fig. 2). EDX of the plates revealed presence of three major elements: carbon, nitrogen, and oxygen, with carbon being the predominant element, consistent with a similar make-up to cholesterol based stones. No calcium was detected from EDX. Stone specimens were subsequently placed in different solvents: ethanol, DI water, DI water + detergent, and trichloromethane. Dissolution was only achieved in the trichloromethane solution.

## 3 Discussion

This case report illustrates a unique presentation of hepatolithiasis for several reasons. While most patients are diagnosed with hepatolithiasis in the 5th or 6th decade of life, presentation in adolescence is rare. Primary considerations in the differential diagnosis include primary sclerosing cholangitis (PSC), recurrent pyogenic cholangitis, bile acid transporter defect, and Caroli disease. PSC is a chronic cholestatic liver disease of unknown etiology characterized by inflammation and fibrosis of intra and extrahepatic bile ducts leading to progressive stricturing and dilatation of the biliary ducts. Given the extensive stone disease and diffuse involvement of the liver specimen, this diagnosis is unlikely. In addition, PSC generally affects the entire biliary tract and is not isolated to one side of the liver as in our patient. Recurrent pyogenic cholangitis, which is endemic in East Asia, remains unlikely given the patient’s lack of travels to that area and Caucasian descent. Moreover, her frequent hospitalizations occurred without systemic signs of infections and her bile was sterile. A bile acid transporter defect, such as ABCB4/MDR3, can present with hepatolithiasis at a younger age, but the absence of significant disease in the right hepatic duct system argues against this as a cause [6]. Caroli disease, a congenital disorder characterized by multifocal, segmental dilatation of large intrahepatic ducts, is a possibility in cases of early-onset hepatolithiasis. However, the patients’ history of MRCP reports did not strongly favor this diagnosis because the biliary dilatation appeared more fusiform than saccular, which would be an abnormal presentation. Moreover, this diagnosis was not supported by final histological examination. Ultimately, we are left with an unknown cause of this patient’s development of early-onset hepatolithiasis. We attribute her problem to a yet undescribed or undiscovered genetic cause given the patient’s strong family history of stone disease. Her history was notable for early gallbladder disease in her father and paternal cousin, aunt and grandmother, which would argue for a genetic component. Genetic testing of family members may help better characterize a cause for this patient’s rare case.

The stone analysis, while not revealing of a definite diagnosis, might help point in the direction of the etiology. The stones collected from this patient were composed primarily of cholesterol. Cholesterol is commonly found as a major component of stones in the extrahepatic ducts and gallbladder but it is rarely found as the main component in intrahepatic stones [7]. In the absence of precipitating factors such as infection, cholestasis, or structural abnormalities which were not apparent in this patient, defects of bile metabolism are likely candidates for relative cholesterol supersaturation and nucleation within the liver ducts [8].

Our patient will need close clinical follow-up as she remains at high risk of developing intrahepatic stone formation in her remant right liver. A life-time of interval liver function tests, and cross-sectional imaging is strongly recommended to detect early recurrent disease to prevent progressive indolent liver injury and ultimately liver failure. Indeed, occasionally in patients with recurrent pyogenic cholangitis, liver transplantation as a salvage therapy is required [9].

## 4 Conclusion

Presentations of early-onset hepatolithiasis is rare, particularly in Western countries. Definitive surgical management is required in complex cases. In cases with unknown etiology, further work-up is indicated and can include stone analysis and genetic testing. If indicated, life-long follow-up should be incorporated, as is the recommended management in this case. A detailed family history, genetic testing, and extensive stone analysis may be performed to help characterize disease etiology in unique presentations.

## Declaration of Competing Interest

The authors declare that they have no conflicts of interest.

## Sources of funding

Jorge Mena received the UCSF PROF PATH Pre-Doctoral Grant to support his research.

## Ethical approval

Ethical approval exempted by our institution.

## Consent

Written informed consent was obtained from the patient for publication of this case report and accompanying images. A copy of the written consent is available for review by the Editor-in-Chief of this journal on request.

## Author contribution

Jonathan Freise: data collection, manuscript writing, design of study.

Jorge Mena: performed stone analysis, manuscript writing and revision.

Kwun Wah Wen: evidence collection, manuscript revision.

Marshall Stoller: supervision of stone analysis, design of study.

Sunita Ho: supervision of stone analysis, design of study, manuscript revision.

Carlos Corvera: managed patient and performed the surgery, design of study, manuscript revision, corresponding author.

## Registration of research studies

1. Name of the registry: n/a.
2. Unique Identifying number or registration ID: n/a.
3. Hyperlink to your specific registration (must be publicly accessible and will be checked).

## Guarantor

Carlos Corvera.

## Provenance and peer review

Not commissioned, externally peer-reviewed.
